# Cystic Fibrosis Mortality Trends 1999–2024—A CDC Wonder Study

**DOI:** 10.3390/arm94040047

**Published:** 2026-07-14

**Authors:** Palak Grover, Rahul Jain, Gurleen Kaur, Niroshan Ranjan, Bipneet Singh

**Affiliations:** 1 Henry Ford Jackson Hospital, Jackson, MI 49201, USA; pgrover1@hfhs.org (P.G.); nranjan1@hfhs.org (N.R.); 2 Sri Manakula Vinayagar Medical College, Puducherry 605107, India; rahul.jainmanohar@gmail.com; 3 Government Medical College, Amritsar 143001, Punjab, India; drgurleenkaur25@gmail.com; 4 University of Kentucky, Lexington, KY 40506, USA

**Keywords:** cystic fibrosis, ivacaftor, trkafta

## Abstract

**Highlights:**

- CF mortality declined by more than half over 26 years, with the age-adjusted mortality rate falling from 0.17 to 0.07 per 100,000 (1999–2024). The steepest reduction coincided temporally with elexacaftor/tezacaftor/ivacaftor availability, though causation cannot be established from ecological data.
- The female-to-male death count ratio shifted from 1.05 to 0.97 across therapeutic periods; however, age-adjusted mortality rates were identical between sexes within each period, and this observation should be considered hypothesis-generating rather than evidence of differential benefit.
- Despite absolute mortality declines in every U.S. Census Region, the South’s share of deaths grew from 37.2% to 41.6%. Several factors may contribute to this pattern, including differences in modulator-eligible genotype prevalence, socioeconomic barriers, and insurance-related limitations, though the current data cannot determine their relative contributions.
- Annual deaths stabilized at approximately 235 per year from 2021 through to 2024, a novel observation that may reflect the approximately 10% of CF patients ineligible for current modulators, irreversible organ damage in older patients, and evolving death certificate coding practices.

**Abstract:**

Cystic fibrosis (CF) is an autosomal recessive disorder caused by mutations in the *CFTR* gene. The sequential approval of CFTR modulators ivacaftor (2012), lumacaftor/ivacaftor (2015), tezacaftor/ivacaftor (2018), and elexacaftor/tezacaftor/ivacaftor (2019) has transformed CF care, but population-level mortality trends across therapeutic periods have not been comprehensively assessed. We conducted a retrospective analysis of CF mortality in the United States from 1999 to 2024 using CDC WONDER Underlying Cause of Death data (ICD-10 codes E84.0–E84.9). Age-adjusted mortality rates (AAMR) per 100,000 were calculated using the 2000 U.S. standard population. The study period was divided into three periods: pre-modulator (1999–2011), early modulator (2012–2018), and elexacaftor/tezacaftor/ivacaftor (2019–2024). Annual mortality trends were evaluated using segmented log-linear Poisson regression, with annual death counts as the outcome and the corresponding U.S. population as an offset. Candidate models with multiple change points were compared to identify distinct temporal segments. Annual percent changes (APCs) and 95% confidence intervals (CIs) were estimated for each segment. Prespecified therapeutic periods, including pre-modulator (1999–2011), early modulator (2012–2018), and ETI period (2019–2024), were retained for descriptive analyses. Trends were stratified by sex and U.S. Census Region. A total of 10,959 CF deaths were recorded over 26 years. In the pre-modulator period, mortality was stable at a mean of 478 deaths/year (AAMR 0.14–0.17). The early modulator period showed a modest 5.4% reduction in mean annual deaths (452/year). The period following ETI availability was associated with a decline to 263/year, a 47% reduction from the pre-modulator period (AAPC −8.6%/year). The AAMR declined from 0.17 (1999) to 0.07 (2024). The female-to-male death count ratio shifted from 1.05 to 0.97 across periods, though age-adjusted rates were identical between sexes within each period, and this finding should be interpreted cautiously. Regionally, the South’s share of CF deaths grew from 37.2% to 41.6% despite absolute declines in all regions, suggesting potential geographic disparities that warrant further investigation with individual-level data. Segmented regression identified change points in 2005 and 2015. Mortality declined significantly from 1999 through 2005 (APC, −2.70%; 95% CI, −4.01% to −1.37%), remained stable from 2006 through 2015 (APC, +0.21%; 95% CI, −0.47% to +0.90%), and declined sharply from 2016 through 2024 (APC, −9.76%; 95% CI, −10.66% to −8.84%). CF mortality in the United States has declined by more than half since the introduction of CFTR modulators. The shift in the sex-based death count ratio and the concentration of remaining deaths in the South are hypothesis-generating observations that require confirmation with individual-level data. These ecological findings cannot establish causation, as concurrent changes in supportive care, lung transplantation practices, and COVID-19 pandemic effects may have contributed to the observed trends.

## 1. Introduction

Cystic Fibrosis is an autosomal recessive disorder caused by mutations in the cystic fibrosis transmembrane conductance regulator (*CFTR*) gene, affecting more than 30,000 individuals in the United States and approximately 89,000 worldwide [1]. Loss of functional CFTR protein leads to multiorgan dysfunction dominated by progressive pulmonary disease, which remains the leading cause of morbidity and mortality, accounting for over 60% of CF-related deaths [2]. Historically, CF was a disease of childhood, with a median survival of approximately 17 years in 1970 [3]. Median survival has since improved dramatically, reaching 53.1 years (95% CI, 51.6–54.7) for individuals born from 2017 through 2021.

The development of CFTR modulator therapies has fundamentally transformed the CF treatment landscape. Ivacaftor, a CFTR potentiator approved in 2012, was the first therapy to target the underlying molecular defect, initially benefiting approximately 4–5% of the CF population with gating mutations [3]. In the landmark STRIVE trial, ivacaftor improved ppFEV_1_ by 10.5 percentage points versus placebo (95% CI, 8.5–12.5%), with a 55% reduction in pulmonary exacerbations (rate ratio 0.43; 95% CI, 0.27–0.68). Subsequent dual-therapy combinations, lumacaftor/ivacaftor (2015) and tezacaftor/ivacaftor (2018), extended modulator eligibility to individuals homozygous for the F508del mutation [4]. Lumacaftor/ivacaftor improved ppFEV_1_ by 2.6 to 4.0 percentage points above placebo (*p* < 0.001) in two parallel phase 3 trials enrolling 1108 patients. Tezacaftor/ivacaftor improved ppFEV_1_ by 4.0 percentage points in F508del homozygotes (EVOLVE) and 6.8 percentage points in F508del/RF heterozygotes (EXPAND), with a 35% reduction in pulmonary exacerbations. The approval of elexacaftor/tezacaftor/ivacaftor (ETI) in October 2019 represented a paradigm shift, as approximately 90% of the CF population became eligible for highly effective modulator therapy (HEMT) [3,5]. Clinical trials demonstrated that ETI improved FEV_1_ and reduced pulmonary exacerbation rates in patients with at least one F508del allele, leading to a reduction in mortality and lung transplantation rates [1,6,7]. Individual-level cohort studies have subsequently demonstrated a 66% lower hazard of death with CFTR modulator initiation (HR 0.34; 95% CI, 0.28–0.41), and real-world registry data show a 78% reduction in mortality relative to the pre-ETI year [8,9,10]. 

Despite these advances, improvements in CF outcomes have not been uniform across demographic and geographic subgroups. A well-documented female survival disadvantage has persisted for decades, attributed to earlier acquisition of Pseudomonas aeruginosa, more frequent pulmonary exacerbations, and hormonal influences on airway inflammation and mucociliary clearance [11,12]. Whether HEMT has narrowed or eliminated this sex-based mortality gap at the population level remains incompletely characterized. Racial, ethnic, and geographic disparities have also been reported. Recent evidence suggests that females with CF continue to experience worse pulmonary morbidity than males even in the HEMT period. Racial, ethnic, and geographic disparities have also been reported; only 69.7% of Black CF patients carry modulator-eligible mutations compared with 92.4% of non-Hispanic White patients. However, these analyses have not fully captured the period following widespread HEMT adoption, which has not been assessed [13].

Prior population-level mortality studies using CDC WONDER data have analyzed CF-related deaths through 2020. Singh et al. (2023) analyzed CF mortality from 1999 to 2020 using joinpoint regression and reported a decline in age-standardized mortality from 1.9 to 1.04 per million (*p* = 0.013), with the median age of death increasing from 24 to 37 years [4]. No study has assessed trends through 2024 using a three-period therapeutic framework aligned with CFTR modulator approval milestones, nor has the observation of mortality stabilization at approximately 235 deaths per year from 2021 onward been previously reported.

## 2. Materials and Methods

### 2.1. Study Design and Data Sources

This was a population-based, retrospective analysis of CF mortality trends in the United States from 1999 through 2024. Mortality data were obtained from the Centers for Disease Control and Prevention Wide-ranging Online Data for Epidemiologic Research (CDC WONDER) system, which compiles death certificate information from all 57 U.S. vital statistics jurisdictions through the Vital Statistics Cooperative Program.

Two CDC WONDER datasets were queried: (1) the Underlying Cause of Death, 1999–2020 dataset (released 2021), which provided mortality data for the first 22 years of the study period; and (2) the Underlying Cause of Death, 2018–2024, Single Race dataset (released 2026), which extended coverage through 2024. The overlapping years of 2019 and 2020 were used to verify cross-dataset concordance; death counts were identical across both sources for these years, confirming consistency.

### 2.2. Case Identification

Deaths attributable to cystic fibrosis were identified using the following International Classification of Diseases, Tenth Revision (ICD-10) codes listed as the underlying cause of death: E84.0 (cystic fibrosis with pulmonary manifestations), E84.1 (cystic fibrosis with intestinal manifestations), E84.8 (cystic fibrosis with other manifestations), and E84.9 (cystic fibrosis, unspecified).

### 2.3. Population Denominators

Population estimates used as denominators for rate calculations varied by dataset. For the 1999–2020 dataset, bridged-race intercensal and postcensal population estimates were used, including revised intercensal estimates for 2001–2009 (released 26 October 2012), 1 April Census counts for 2000 and 2010, and postcensal estimates for remaining years. For the 2018–2024 dataset, single-race population estimates were used, based on the Modified Blended Base produced by the U.S. Census Bureau instead of the 1 April 2020, decennial population count for years 2020 and later. Population estimates for 2022–2024 were derived from Vintage 2022, 2023, and 2024 postcensal series, respectively.

### 2.4. Rate Calculations

Crude mortality rates were calculated as the number of cystic fibrosis deaths divided by the corresponding population, expressed per 100,000 persons. Age-adjusted mortality rates were calculated using the direct method, standardized to the 2000 U.S. Standard Population, as implemented by CDC WONDER. Both crude and age-adjusted rates were reported with 95% confidence intervals.

### 2.5. Stratification and Subgroup Analyses

Mortality data were stratified by year, U.S. Census Region (Northeast, Midwest, South, West), and sex. National trends were assessed using annual death counts and age-adjusted rates. 

Annual cystic fibrosis mortality trends were evaluated using segmented log-linear regression. Because age-adjusted mortality rates exported from CDC WONDER were rounded at low absolute rate values, the primary trend model used annual death counts with the corresponding U.S. population included as an offset. This approach preserved the full information contained in the annual mortality counts while accounting for changes in the underlying population over time. Models assumed a Poisson distribution with a log link.

Candidate segmented models containing zero, one, two, or three change points were evaluated. Change points were restricted to interior years to ensure an adequate number of annual observations within each segment. The optimal model was selected based on model fit and parsimony. For each segment, the annual percent change (APC) was calculated as 100 × (e^β^ − 1), where β is the estimated annual change in the log mortality rate. Two-sided 95% confidence intervals (CIs) and *p* values were calculated for each APC. An APC was considered statistically significant when its 95% CI excluded zero and *p* < 0.05 [14]. 

The average annual percent change (AAPC) for the full 1999–2024 study period was calculated as a duration-weighted geometric average of the segment-specific APC estimates.

Because the introduction of elexacaftor/tezacaftor/ivacaftor in 2019 represented a prespecified clinically relevant intervention, an interrupted time-series analysis was additionally performed as a sensitivity analysis. The model included terms for the underlying pre-intervention temporal trend, an immediate level change beginning in 2019, and a change in the post-2019 slope. Effect estimates were exponentiated and reported as incidence rate ratios (IRRs) with 95% CIs. The level-change term estimated the immediate change in mortality associated with entry into the elexacaftor/tezacaftor/ivacaftor period, whereas the interaction term estimated the additional annual change in slope after 2019 [15].

The three prespecified therapeutic periods, including pre-modulator (1999–2011), early modulator (2012–2018), and elexacaftor/tezacaftor/ivacaftor period (2019–2024), were retained for descriptive comparisons of mean annual deaths and age-adjusted mortality rates. These period-based comparisons were interpreted as descriptive and were not used to determine the locations of statistical change points.

### 2.6. Outcome Definitions

CF-attributable mortality rate: Age-adjusted mortality rate per 100,000 population for deaths with CF listed as the underlying cause of death (ICD-10 codes E84.0, E84.1, E84.8, E84.9), standardized to the 2000 U.S. standard population.

### 2.7. Crude Mortality Rate: Deaths per 100,000 Population, Unadjusted for Age

Average annual percent change (AAPC): Estimated from the slope coefficient of the segmented regression model within each therapeutic period (see Statistical Approach below), reported with 95% confidence intervals.

Mortality rate ratio (MRR): The ratio of age-adjusted mortality rates between subgroups (e.g., female vs. male, or between therapeutic periods), estimated using Poisson regression with 95% confidence intervals. 

### 2.8. Stratification of Therapeutic Periods

To assess the temporal association between the introduction of CFTR modulator therapies and population-level mortality, the study period was divided into three periods defined by regulatory milestones:

1. Pre-modulator period (1999–2011): The period preceding the approval of any CFTR modulator therapy, serving as the baseline comparator.
2. Early CFTR modulator period (2012–2018): Beginning with the approval of ivacaftor (January 2012, targeting gating mutations in approximately 4–5% of patients), encompassing the approval of lumacaftor/ivacaftor (July 2015, extending eligibility to F508del homozygotes, approximately 25% of patients), and ending with the approval of tezacaftor/ivacaftor (February 2018).
3. Elexacaftor/tezacaftor/ivacaftor period (2019–2024): Beginning with the approval of elexacaftor/tezacaftor/ivacaftor (Trikafta) in October 2019, which extended modulator eligibility to patients with at least one F508del allele (approximately 90% of the CF population). Although the drug was approved in late 2019, the full calendar year of 2019 was included in this period to capture the transitional period and because partial-year effects were expected.

### 2.9. Statistical Approach

Aggregate trends were assessed by comparing mean annual death counts, crude rates, and age-adjusted rates across the three therapeutic periods. Percent changes in mortality were calculated between periods using both absolute death counts and age-adjusted rates. 

Formal joinpoint regression was not performed for several reasons. First, the therapeutic eras were defined a priori based on FDA approval dates, rendering data-driven identification of inflection points in trends unnecessary. Second, the limited number of annual observations within the post-Trikafta period (n = 6) constrained the statistical power of segmented regression models to detect sub-trends.

## 3. Results

Table 1 and Table 2 demonstrate the year-wise deaths, crude death rate, and predefined period-wise mean age-adjusted rates, respectively. Minor differences in period totals across tables reflect CDC WONDER cell suppression rules applied to stratified queries. 

During the 13 years preceding the availability of CFTR modulator therapy, mortality remained essentially stable. A total of 6216 deaths were recorded, with a mean of 478 deaths per year. The age-adjusted mortality rate fluctuated between 0.14 and 0.17 per 100,000 population (mean: 0.158 per 100,000).

The sequential approvals of ivacaftor in 2012, lumacaftor/ivacaftor in 2015, and tezacaftor/ivacaftor in 2018 introduced the first disease-modifying therapies for cystic fibrosis. During these seven years, 3166 deaths were recorded, with a mean of 452 deaths per year, a modest 5.4% reduction from the pre-modulator period mean. 

The period following ETI availability (2019–2024) was associated with a substantial reduction in mortality. A total of 1577 deaths were recorded over six years, with a mean of 263 deaths per year representing a 47% reduction from the pre-modulator period mean, and a 42% reduction from the early modulator period mean.

### 3.1. Sex Variation

The F: M mortality ratio is demonstrated in Table 3. However, the female-to-male ratio stayed consistent.

### 3.2. Regional Variation

Table 4 demonstrates the regional trends in CF mortality based on drug period.

### 3.3. Temporal Trend Analysis

Segmented log-linear regression identified two change points in the national cystic fibrosis mortality trend, occurring in 2005 and 2015. The resulting model divided the 1999–2024 study period into three distinct temporal segments.

From 1999 through 2005, cystic fibrosis mortality declined significantly, with an APC of −2.7% per year (95% CI, −4.5% to –0.8%; *p* = 0.004). This initial decline was followed by a period of relative stability from 2006 through 2015, during which no statistically significant temporal change was observed (APC, +0.2% per year; 95% CI, −0.7% to +1.2%; *p* = 0.66). Beginning in 2016, mortality entered a period of pronounced and sustained decline. From 2016 through 2024, the mortality rate decreased by 9.8% annually (95% CI, −11.0% to −8.5%; *p* < 0.001). Across the entire 1999–2024 study period, the estimated AAPC was approximately −4.1% per year.

The empirically identified change points did not correspond precisely to the prespecified regulatory milestones of initial ivacaftor approval in 2012 or elexacaftor/tezacaftor/ivacaftor approval in late 2019. Instead, the model identified the onset of the steepest sustained decline in 2016, temporally coinciding with the period of expanding CFTR modulator eligibility following the introduction of lumacaftor/ivacaftor and preceding the subsequent availability of highly effective triple-modulator therapy.

### 3.4. Interrupted Time-Series Sensitivity Analysis

In the prespecified interrupted time-series analysis centered on the 2019 introduction of elexacaftor/tezacaftor/ivacaftor, entry into the post-2019 period was associated with an immediate reduction in cystic fibrosis mortality. The estimated level-change IRR was 0.74, corresponding to an approximately 26% reduction in mortality relative to the counterfactual continuation of the preceding trend. The post-2019 period was also associated with an additional annual decline of approximately 8.0% relative to the pre-intervention slope.

**Figure 1:**
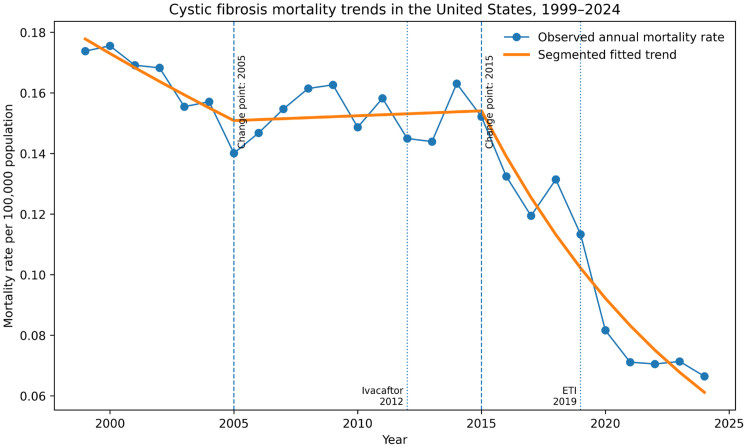
The figure shows the data-derived change points at 2005 and 2015 and separately marks the 2012 ivacaftor and 2019 ETI milestones. The figure was generated using generative AI, ChatGPT Plus version 5.5.

These findings (Figure 1) were consistent with the descriptive mortality pattern. Annual deaths decreased from 430 in 2018 to 372 in 2019 and 269 in 2020, reaching 236 in 2021. Mortality subsequently remained at a substantially lower level, with 235 deaths in 2022, 239 in 2023, and 226 in 2024. Thus, although the data-driven segmented model identified the beginning of the steepest sustained mortality decline in 2016, the interrupted time-series analysis supported an additional reduction associated with the elexacaftor/tezacaftor/ivacaftor period.

Over the 26-year study period, the age-adjusted CF mortality rate declined from 0.17 per 100,000 (1999) to 0.07 per 100,000 (2024), corresponding to an overall AAPC of −3.5% per year as demonstrated in Table 5.

## 4. Discussion

In this population-based analysis of 10,959 cystic fibrosis (CF) deaths in the United States from 1999 through 2024, mortality declined substantially but nonlinearly over time. Segmented log-linear Poisson regression identified change points in 2005 and 2015. Mortality declined from 1999 through 2005 (APC, −2.70% per year; 95% CI, −4.01% to −1.37%), was stable from 2006 through 2015 (APC, +0.21%; 95% CI, −0.47% to +0.90%), and then declined sharply from 2016 through 2024 (APC, −9.76%; 95% CI, −10.66% to −8.84%). These empirically identified inflection points did not coincide exactly with the 2012 approval of ivacaftor or the late-2019 approval of elexacaftor/tezacaftor/ivacaftor (ETI), underscoring that the observed population trend cannot be attributed to a single therapeutic milestone. Rather, the findings are most consistent with a temporal association between progressively expanding CFTR modulator availability and declining CF mortality, occurring alongside concurrent advances in multidisciplinary care, antimicrobial therapy, nutrition, transplantation practice, and other secular changes [1,2,3,4,5,6,7].

The early modulator era should therefore be interpreted in the context of the trend analysis rather than as a sequence of clinical-trial summaries. Mean annual deaths declined only modestly from 478 in 1999–2011 to 452 in 2012–2018, while the mean age-adjusted mortality rate decreased from 0.158 to 0.142 per 100,000. The segmented model showed no significant change from 2006 through 2015, followed by the onset of a steep sustained decline in 2016. This timing is compatible with a cumulative population effect as modulator eligibility expanded beyond the small subgroup initially eligible for ivacaftor to larger groups receiving dual-modulator therapy, but the ecological design does not permit attribution of the 2016 change point to a specific drug or exposure [3,16,17,18,19,20,21,22,23].

The period following ETI availability was associated with the lowest mortality observed in the study. Mean annual deaths fell to 263 during 2019–2024, and the age-adjusted mortality rate declined from 0.11 per 100,000 in 2019 to 0.07 in 2024. The interrupted time-series sensitivity analysis estimated an immediate level-change incidence rate ratio of 0.74, corresponding to an approximately 26% reduction relative to the counterfactual continuation of the preceding trend, with an additional post-2019 annual decline of approximately 8%. These findings strengthen the evidence that the mortality trajectory changed during the ETI period; however, they remain associational. Individual-level studies and registry data reporting improved survival after modulator initiation provide biologic and clinical plausibility, but CDC WONDER contains no information on genotype, treatment exposure, adherence, duration of therapy, disease severity, or socioeconomic status [3,5,6,7,21,24,25,26].

COVID-19 is an important potential confounder because the steepest single-year decline occurred from 2019 to 2020, when annual deaths fell from 372 to 269 (27.7%). During the pandemic, many people with CF adopted intensive shielding and infection-prevention behaviors that may have reduced exposure not only to SARS-CoV-2 but also to other respiratory pathogens. Healthcare delivery and utilization also changed substantially, and national death-certificate practices were affected during the pandemic. Consequently, the 2020 decline cannot be interpreted as an isolated pharmacologic effect of ETI. At the same time, mortality remained low after the first pandemic year, with 236 deaths in 2021, 235 in 2022, 239 in 2023, and 226 in 2024, arguing against a purely transient 2020 phenomenon. The interrupted time-series analysis should therefore be viewed as supportive of an additional change during the ETI period, while residual confounding by pandemic-related behavioral, healthcare, and coding changes remains unavoidable in an ecological study [27,28].

The stabilization of annual CF deaths at approximately 235 per year from 2021 through 2024 is among the most clinically important observations of this analysis. Several nonexclusive mechanisms may contribute to this apparent mortality floor. First, a minority of people with CF remain ineligible for currently available modulators because of nonresponsive mutations, including some premature-termination and splice variants, while others discontinue therapy because of intolerance [26,29]. Second, restoration of CFTR function may not fully reverse advanced bronchiectasis, chronic infection, or established extrapulmonary organ damage; therefore, patients entering the highly effective modulator era with advanced disease may retain substantial residual risk [3,7,26]. Third, as survival improves, the CF population is aging, and the relative contribution of CF-related diabetes, cardiovascular risk factors, malignancy, and other age-associated conditions may increase. The recent observation that mortality did not decline in the oldest age group is consistent with this possibility [5]. Fourth, CF may increasingly be recorded as a contributing rather than underlying cause of death in older patients with multimorbidity, potentially altering ascertainment in an underlying-cause analysis. These explanations are hypotheses rather than mechanisms demonstrated by the present data.

The approval of vanzacaftor/tezacaftor/deutivacaftor in late 2024 introduces another therapeutic milestone beyond the observation window [30]. Its once-daily regimen and activity across additional responsive CFTR variants may improve treatment options for some patients, although whether it will lower the apparent post-2021 mortality plateau cannot yet be determined. Future analyses extending beyond 2024 and studies using individual-level CF Foundation Patient Registry data will be needed to determine whether the plateau persists and to characterize the genotypes, treatment exposures, comorbidities, and causes of death among the remaining decedents.

Lung transplantation is another important competing event in interpreting CF mortality trends. ETI availability has been accompanied by marked reductions in lung-transplant referrals, wait-listing, and transplantation among people with CF [3,26,31]. This may reflect fewer patients progressing to end-stage lung disease, but transplantation also changes subsequent cause-of-death coding: deaths after transplantation may be attributed to transplant complications, infection, malignancy, or other causes rather than CF as the underlying cause. The direction and magnitude of this effect cannot be determined from CDC WONDER. Therefore, changing transplantation practices may contribute to the observed temporal pattern and should not be treated solely as a downstream consequence of modulator therapy.

The sex-stratified findings do not demonstrate a statistically significant differential mortality benefit. Although the female-to-male death-count ratio shifted from a slight female excess to a slight male excess across therapeutic periods, the female-to-male mortality rate ratios were 0.990 (95% CI, 0.941–1.041), 0.981 (95% CI, 0.915–1.052), and 0.947 (95% CI, 0.858–1.045) in the pre-modulator, early-modulator, and ETI periods, respectively; all confidence intervals included 1.0 and all *p*-values were nonsignificant. Thus, the historical female survival disadvantage cannot be considered reversed on the basis of these data. The small shift in raw proportional representation is hypothesis-generating and may reflect changes in the composition of deaths as overall mortality declines rather than a sex-specific pharmacologic effect [11,12,32,33,34,35].

The regional analysis also requires cautious interpretation. Population-adjusted mortality rates declined in every U.S. Census Region. In the ETI period, the South had the numerically highest regional mortality rate (0.085 per 100,000) compared with the Northeast (0.077), Midwest (0.072), and West (0.076), but its mortality rate ratio versus the Northeast was not statistically significant (MRR, 1.111; 95% CI, 0.963–1.283; *p* = 0.150). The South’s increasing proportional share of deaths, from 37.2% to 41.6%, therefore should not be interpreted by itself as evidence of worsening regional mortality or impaired modulator access. Proportional shares are sensitive to regional population size and to different rates of decline elsewhere. Prior literature identifies geographic variation in CF outcomes and racial and ethnic differences in modulator eligibility, but the present study did not measure genotype distribution, insurance coverage, socioeconomic conditions, treatment access, or CF prevalence by region [29,36]. These factors remain plausible hypotheses for future study rather than explanations established by our analysis.

Race and ethnicity were not included as primary subgroup analyses. CF deaths are uncommon in several racial and ethnic strata, increasing the risk of unstable estimates and CDC WONDER cell suppression. In addition, this study spans the 1999–2020 bridged-race dataset and the 2018–2024 single-race dataset, which use different population-denominator methodologies and create additional comparability concerns for race-stratified longitudinal analyses. This omission is important because racial and ethnic disparities in diagnosis, genotype distribution, modulator eligibility, access to specialized care, and outcomes remain clinically relevant [29,32,36]. Individual-level registry studies are better suited to evaluate these relationships while accounting for genotype, treatment exposure, socioeconomic factors, and disease severity.

These findings should be interpreted within the limitations of an ecological mortality study. CF was identified only when listed as the underlying cause of death, so deaths in which CF was a contributing cause may have been missed. Misclassification and temporal changes in death-certificate coding are possible. CDC WONDER does not provide individual-level information on *CFTR* genotype, modulator eligibility, actual treatment exposure, adherence, treatment duration, lung function, transplantation, socioeconomic status, insurance, comorbidities, or other markers of disease severity. The analysis cannot separate the effects of modulator therapies from concurrent improvements in CF care or from pandemic-related changes. Although segmented regression and interrupted time-series analyses provide formal statistical evaluation of changes in temporal slope and level, they do not establish causation, and the 2019 intervention point is complicated by late-year approval and rapid but incomplete uptake. Finally, subgroup analyses may be affected by small numbers and suppression, and the use of two overlapping CDC WONDER datasets introduces potential denominator comparability issues despite concordant death counts in overlapping years [15]. 

In conclusion, U.S. CF mortality declined by more than half from 1999 through 2024, with a statistically significant acceleration beginning in 2016 and an additional reduction temporally associated with the period following ETI availability. The persistence of approximately 226–239 deaths annually from 2021 through 2024 suggests a possible mortality floor that warrants continued surveillance. The sex-based shift was not statistically significant, and regional differences should be interpreted using population-adjusted rates rather than proportional death counts alone. These results demonstrate temporal associations, not treatment effects at the individual level. Future registry-linked studies should determine which patients remain at greatest risk by integrating genotype, modulator exposure, adherence, transplantation, race and ethnicity, socioeconomic factors, and competing causes of death.

## Figures and Tables

**Table 1:** National CF Mortality Trends: Age-Adjusted Rates per 100,000 (1999–2024).

Year	Deaths	Population	Crude Rate	Age-Adjusted Rate	95% CI
1999	485	279,040,168	0.17	0.17	0.15–0.19
2000	494	281,421,906	0.18	0.17	0.15–0.19
2001	482	284,968,955	0.17	0.17	0.15–0.18
2002	484	287,625,193	0.17	0.17	0.15–0.18
2003	451	290,107,933	0.16	0.16	0.14–0.17
2004	460	292,805,298	0.16	0.16	0.14–0.17
2005	414	295,516,599	0.14	0.14	0.13–0.16
2006	438	298,379,912	0.15	0.15	0.13–0.16
2007	466	301,231,207	0.15	0.15	0.14–0.17
2008	491	304,093,966	0.16	0.16	0.15–0.18
2009	499	306,771,529	0.16	0.16	0.15–0.18
2010	459	308,745,538	0.15	0.15	0.14–0.16
2011	493	311,591,917	0.16	0.16	0.15–0.17
2012	455	313,914,040	0.14	0.15	0.13–0.16
2013	455	316,128,839	0.14	0.15	0.13–0.16
2014	520	318,857,056	0.16	0.16	0.15–0.18
2015	489	321,418,820	0.15	0.15	0.14–0.17
2016	428	323,127,513	0.13	0.13	0.12–0.15
2017	389	325,719,178	0.12	0.12	0.11–0.13
2018	430	327,167,434	0.13	0.13	0.12–0.15
2019	372	328,239,523	0.11	0.11	0.10–0.13
2020	269	329,484,123	0.08	0.08	0.07–0.09
2021	236	331,893,745	0.07	0.07	0.06–0.08
2022	235	333,287,557	0.07	0.07	0.06–0.08
2023	239	334,914,895	0.07	0.07	0.06–0.08
2024	226	340,110,988	0.07	0.07	0.06–0.08

**Table 2:** AAR for CF, stratified by treatment periods.

Period	Years	Total Deaths	Mean Annual Deaths	Mean Age-Adjusted Rate	Rate Range
(1999–2011)	1999–2011	6216	478	0.158 per 100,000	0.14–0.17
(2012–2018)	2012–2018	3166	452	0.142 per 100,000	0.12–0.16
(2019–2024)	2019–2024	1577	263	0.078 per 100,000	0.07–0.11

**Table 3:** Sex distribution of CF mortality.

Therapeutic Period	Female Deaths, n	Female Mortality Rate per 100,000 (95% CI)	Male Deaths, n	Male Mortality Rate per 100,000 (95% CI)	Female-to-Male MRR (95% CI)	*p*-Value
Pre-modulator (1999–2011)	3078	0.158 (0.152–0.164)	3015	0.160 (0.155–0.166)	0.990 (0.941–1.041)	0.691
Early modulator (2012–2018)	1867	0.140 (0.134–0.147)	1862	0.143 (0.137–0.150)	0.981 (0.915–1.052)	0.587
ETI period (2019–2024)	519	0.064 (0.058–0.069)	518	0.067 (0.062–0.073)	0.947 (0.858–1.045)	0.281

**Table 4:** Regional distribution of CF mortality.

Period	Region	Mortality Rate/100k	MRR vs. Northeast	95% CI	*p*
Pre-modulator	Northeast	0.164	Reference	–	–
Pre-modulator	Midwest	0.176	1.076	0.996–1.161	0.063
Pre-modulator	South	0.162	0.991	0.923–1.063	0.797
Pre-modulator	West	0.135	0.822	0.758–0.892	<0.001
Early modulator	Northeast	0.137	Reference	–	–
Early modulator	Midwest	0.146	1.066	0.952–1.193	0.268
Early modulator	South	0.15	1.093	0.988–1.209	0.084
Early modulator	West	0.126	0.917	0.819–1.028	0.136
ETI period	Northeast	0.077	Reference	–	–
ETI period	Midwest	0.072	0.943	0.798–1.113	0.487
ETI period	South	0.085	1.111	0.963–1.283	0.15
ETI period	West	0.076	0.999	0.852–1.171	0.988

**Table 5:** Segmented regression analysis of cystic fibrosis mortality trends in the United States, 1999–2024.

Time Period	APC, % per Year	95% CI	*p*-Value
1999–2005	−2.7%/year	−4.01% to −1.37%	0.004
2006–2015	0.21%/year	−0.47% to +0.90%	0.66
2016–2024	−9.76%/year	−10.66% to −8.84%	<0.001

## Data Availability

The raw data supporting the conclusions of this article will be made available by the authors on request.

## References

[1] Cystic Fibrosis Foundation (2022). Patient Registry Annual Data Report.

[2] Burgel P.R., Burnet E., Regard L., Martin C. (2023). The Changing Epidemiology of Cystic Fibrosis: The Implications for Adult Care. Chest.

[3] Ong T., Ramsey B.W. (2023). Cystic Fibrosis: A Review. JAMA.

[4] Singh H., Jani C., Marshall D.C., Franco R., Bhatt P., Podder S., Shalhoub J., Kurman J.S., Nanchal R., Uluer A.Z. (2023). Cystic Fibrosis-Related Mortality in the United States from 1999 to 2020: An Observational Analysis of Time Trends and Disparities. Sci. Rep..

[5] Abraha H.E., Thoma M., Boghossian N.S. (2025). Mortality Rate Trends for Sickle Cell Disease and Cystic Fibrosis in the US. JAMA Pediatr..

[6] Middleton P.G., Mall M.A., Dřevínek P., Lands L.C., McKone E.F., Polineni D., Ramsey B.W., Taylor-Cousar J.L., Tullis E., Vermeulen F. (2019). Elexacaftor-Tezacaftor-Ivacaftor for Cystic Fibrosis with a Single Phe508del Allele. N. Engl. J. Med..

[7] Daines C.L., Tullis E., Costa S., Linnemann R.W., Mall M.A., McKone E.F., Polineni D., Quon B.S., Ringshausen F.C., Rowe S.M. (2023). Long-Term Safety and Efficacy of Elexacaftor/Tezacaftor/Ivacaftor in People with Cystic Fibrosis and at Least One F508del Allele: 144-Week Interim Results from a 192-Week Open-Label Extension Study. Eur. Respir. J..

[8] Wainwright C.E., Elborn J.S., Ramsey B.W., Marigowda G., Huang X., Cipolli M., Colombo C., Davies J.C., De Boeck K., Flume P.A. (2015). Lumacaftor–Ivacaftor in Patients with Cystic Fibrosis Homozygous for Phe508del CFTR. N. Engl. J. Med..

[9] Kurgansky K.E., Collaco J.M., Ng D.K., Lesko C.R. (2026). Effectiveness of Cystic Fibrosis Transmembrane Conductance Regulator Modulator Therapy on Risk of Death for Individuals with Cystic Fibrosis. Chest.

[10] Bower J.K., Volkova N., Ahluwalia N., Sahota G., Xuan F., Chin A., Weinstock T.G., Ostrenga J., Elbert A. (2023). Real-World Safety and Effectiveness of Elexacaftor/Tezacaftor/Ivacaftor in People with Cystic Fibrosis: Interim Results of a Long-Term Registry-Based Study. J. Cyst. Fibros..

[11] Harness-Brumley C.L., Elliott A.C., Rosenbluth D.B., Raghavan D., Jain R. (2014). Gender Differences in Outcomes of Patients with Cystic Fibrosis. J. Women’s Health.

[12] Sweezey N.B., Ratjen F. (2014). The Cystic Fibrosis Gender Gap: Potential Roles of Estrogen. Pediatr. Pulmonol..

[13] Wang A., Lee M., Keller A., Jian S., Lowe K., Finklea J.D., Jain R. (2024). Sex Differences in Outcomes of People with Cystic Fibrosis Treated with Elexacaftor/Tezacaftor/Ivacaftor. J. Cyst. Fibros..

[14] Hincapie-Castillo J.M., Goodin A. (2023). Using Joinpoint Regression for Drug Utilization Research: Tutorial and Case Study of Prescription Opioid Use in the United States. Pharmacoepidemiol. Drug Saf..

[15] Bernal J.L., Cummins S., Gasparrini A. (2017). Interrupted Time Series Regression for the Evaluation of Public Health Interventions: A Tutorial. Int. J. Epidemiol..

[16] Ramsey B.W., Davies J., McElvaney N.G., Tullis E., Bell S.C., Dřevínek P., Griese M., McKone E.F., Wainwright C.E., Konstan M.W. (2011). A CFTR Potentiator in Patients with Cystic Fibrosis and the G551D Mutation. N. Engl. J. Med..

[17] Skilton M., Krishan A., Patel S., Sinha I.P., Southern K.W. (2019). Potentiators (Specific Therapies for Class III and IV Mutations) for Cystic Fibrosis. Cochrane Database Syst. Rev..

[18] Deeks E.D. (2013). Ivacaftor: A Review of Its Use in Patients with Cystic Fibrosis. Drugs.

[19] McKone E.F., Borowitz D., Drevinek P., Griese M., Konstan M.W., Wainwright C., Ratjen F., Sermet-Gaudelus I., Plant B., Munck A. (2014). Long-Term Safety and Efficacy of Ivacaftor in Patients with Cystic Fibrosis Who Have the Gly551Asp-CFTR Mutation: A Phase 3, Open-Label Extension Study (PERSIST). Lancet Respir. Med..

[20] Guimbellot J.S., Baines A., Paynter A., Heltshe S.L., VanDalfsen J., Jain M., Rowe S.M., Sagel S.D. (2021). Long-Term Clinical Effectiveness of Ivacaftor in People with the G551D CFTR Mutation. J. Cyst. Fibros..

[21] Merlo C.A., Thorat T., DerSarkissian M., McGarry L.J., Nguyen C., Gu Y.M., Healy J., Rubin J.L., Brookhart M.A. (2024). Long-Term Impact of Ivacaftor on Mortality Rate and Health Outcomes in People with Cystic Fibrosis. Thorax.

[22] Taylor-Cousar J.L., Munck A., McKone E.F., Van Der Ent C.K., Moeller A., Simard C., Wang L.T., Ingenito E.P., McKee C., Lu Y. (2017). Tezacaftor-Ivacaftor in Patients with Cystic Fibrosis Homozygous for Phe508del. N. Engl. J. Med..

[23] Rowe S.M., Daines C., Ringshausen F.C., Kerem E., Wilson J., Tullis E., Nair N., Simard C., Han L., Ingenito E.P. (2017). Tezacaftor-Ivacaftor in Residual-Function Heterozygotes with Cystic Fibrosis. N. Engl. J. Med..

[24] Heijerman H.G.M., McKone E.F., Downey D.G., Van Braeckel E., Rowe S.M., Tullis E., Mall M.A., Welter J.J., Ramsey B.W., McKee C.M. (2019). Efficacy and Safety of the Elexacaftor Plus Tezacaftor Plus Ivacaftor Combination Regimen in People with Cystic Fibrosis Homozygous for the F508del Mutation: A Double-Blind, Randomised, Phase 3 Trial. Lancet.

[25] Sutharsan S., McKone E.F., Downey D.G., Duckers J., MacGregor G., Tullis E., Van Braeckel E., Wainwright C.E., Watson D., Ahluwalia N. (2022). Efficacy and Safety of Elexacaftor Plus Tezacaftor Plus Ivacaftor Versus Tezacaftor Plus Ivacaftor in People with Cystic Fibrosis Homozygous for F508del-CFTR: A 24-Week, Multicentre, Randomised, Double-Blind, Active-Controlled, Phase 3b Trial. Lancet Respir. Med..

[26] Taylor-Cousar J.L., Robinson P.D., Shteinberg M., Downey D.G. (2023). CFTR Modulator Therapy: Transforming the Landscape of Clinical Care in Cystic Fibrosis. Lancet.

[27] Naehrlich L., Orenti A., Dunlevy F., Kasmi I., Harutyunyan S., Pfleger A., Keegan S., Daneau G., Petrova G., Tješić-Drinković D. (2021). Incidence of SARS-CoV-2 in People with Cystic Fibrosis in Europe Between February and June 2020. J. Cyst. Fibros..

[28] Bain R., Cosgriff R., Zampoli M., Elbert A., Burgel P.-R., Carr S.B., Castaños C., Colombo C., Corvol H., Faro A. (2021). Clinical Characteristics of SARS-CoV-2 Infection in Children with Cystic Fibrosis: An International Observational Study. J. Cyst. Fibros..

[29] McGarry M.E., McColley S.A. (2021). Cystic Fibrosis Patients of Minority Race and Ethnicity Less Likely Eligible for CFTR Modulators Based on CFTR Genotype. Pediatr. Pulmonol..

[30] Keating C., Yonker L.M., Vermeulen F., Prais D., Linnemann R.W., Trimble A., Kotsimbos T., Mermis J., Braun A.T., O’Carroll M. (2025). Vanzacaftor-Tezacaftor-Deutivacaftor Versus Elexacaftor-Tezacaftor-Ivacaftor in Individuals with Cystic Fibrosis Aged 12 Years and Older: Results from Two Randomised, Active-Controlled, Phase 3 SKYLINE Trials. Lancet Respir. Med..

[31] Dhôte T., Grenet D., Reynaud-Gaubert M., Chatron E., Renaud-Picard B., Tissot A., Macey J., Murris-Espin M., Bunel V., Sermet-Gaudelus I. (2026). The Impact of Progressive Expansion of Elexacaftor-Tezacaftor-Ivacaftor Availability on Lung Transplant for Cystic Fibrosis in France. J. Cyst. Fibros..

[32] Montemayor K., Jain R. (2022). Cystic Fibrosis: Highly Effective Targeted Therapeutics and the Impact on Sex and Racial Disparities. Med. Clin. N. Am..

[33] MacKenzie T., Gifford A.H., Sabadosa K.A., Quinton H.B., Knapp E.A., Goss C.H., Marshall B.C. (2014). Longevity of Patients with Cystic Fibrosis in 2000 to 2010 and Beyond: Survival Analysis of the Cystic Fibrosis Foundation Patient Registry. Ann. Intern. Med..

[34] Stephenson A.L., Tom M., Berthiaume Y., Singer L.G., Aaron S.D., Whitmore G., Stanojevic S. (2015). A Contemporary Survival Analysis of Individuals with Cystic Fibrosis: A Cohort Study. Eur. Respir. J..

[35] Nick J.A., Chacon C.S., Brayshaw S.J., Jones M.C., Barboa C.M., Clair C.G.S., Young R.L., Nichols D.P., Janssen J.S., Huitt G.A. (2010). Effects of Gender and Age at Diagnosis on Disease Progression in Long-Term Survivors of Cystic Fibrosis. Am. J. Respir. Crit. Care Med..

[36] Kopp B.T., Nicholson L., Paul G., Tobias J., Ramanathan C., Hayes D. (2015). Geographic Variations in Cystic Fibrosis: An Analysis of the U.S. CF Foundation Registry. Pediatr. Pulmonol..

